# Evaluating imaging repeatability of fully self-service fundus photography within a community-based eye disease screening setting

**DOI:** 10.1186/s12938-024-01222-2

**Published:** 2024-03-12

**Authors:** Juzhao Zhang, Xuan Luo, Deshang Li, Yajun Peng, Guiling Gao, Liangwen Lei, Meng Gao, Lina Lu, Yi Xu, Tao Yu, Senlin Lin, Yingyan Ma, Chunxia Yao, Haidong Zou

**Affiliations:** 1 Shanghai Eye Disease Prevention & Treatment Center/Shanghai Eye Hospital, School of Medicine, Tongji University, Shanghai, China; 2 National Clinical Research Center for Eye Diseases, Shanghai, China; 3 Songjiang Disease Control and Prevention Center, Shanghai, China; 4 Sijing Community Health Service Center, Shanghai, China; 5 Shanghai Engineering Center of Precise Diagnosis and Treatment of Eye Diseases, Shanghai, China; 6 Department of Ophthalmology, Shanghai General Hospital, School of Medicine, Shanghai Jiao Tong University, Shanghai, China

**Keywords:** Fully self-service fundus photography, Imaging repeatability, Image quantitative analysis, Community eye screening, Real-world evidence

## Abstract

**Purpose:**

This study aimed to investigate the imaging repeatability of self-service fundus photography compared to traditional fundus photography performed by experienced operators.

**Design:**

Prospective cross-sectional study.

**Methods:**

In a community-based eye diseases screening site, we recruited 65 eyes (65 participants) from the resident population of Shanghai, China. All participants were devoid of cataract or any other conditions that could potentially compromise the quality of fundus imaging. Participants were categorized into fully self-service fundus photography or traditional fundus photography group. Image quantitative analysis software was used to extract clinically relevant indicators from the fundus images. Finally, a statistical analysis was performed to depict the imaging repeatability of fully self-service fundus photography.

**Results:**

There was no statistical difference in the absolute differences, or the extents of variation of the indicators between the two groups. The extents of variation of all the measurement indicators, with the exception of the optic cup area, were below 10% in both groups. The Bland–Altman plots and multivariate analysis results were consistent with results mentioned above.

**Conclusions:**

The image repeatability of fully self-service fundus photography is comparable to that of traditional fundus photography performed by professionals, demonstrating promise in large-scale eye disease screening programs.

**Supplementary Information:**

The online version contains supplementary material available at 10.1186/s12938-024-01222-2.

## Introduction

Visual impairment emerges as a critical public health concern, imparting substantial impacts on economic sustainability, daily functionality, and mortality rates [1]. Fundus disease is one of the main blinding diseases [2]. Research indicates that screening for fundus diseases, including age-related macular degeneration (AMD) and diabetic retinopathy (DR), is vital in preventing visual impairments [3]. Such screenings have proven to be useful and cost-effective in both low- and high-income countries [4–7]. However, given the limitations pertaining to a scarcity of ophthalmologists, resource shortages, and heavy workload, the present status of eye disease screening is less than ideal [8].

Fundus imaging stands as a fundamental screening approach, providing insight into ocular conditions and systemic diseases [9]. The implementation of remote ophthalmic care and the development of artificial intelligence (AI)-assisted software greatly depend on digitalized fundus images [10–12]. However, in real-world scenarios, diverse societal and environmental factors adversely affect data quality, engendering a notable decline in AI model performance and patient experience [13]. This data quality gap remains a chief obstacle to the extensive adoption of related advancements in clinical environments.

As an engineering innovation to traditional fundus imaging devices, fully automatic fundus cameras may help address this issue. Incorporating automation technology into fundus cameras empowers patients or non-ophthalmic specialists to perform fundus photography, without the need for professional technician involvement. This method holds certain potential for preliminary screening and monitoring of fundus lesions in resource-limited medical scenarios [14]. Furthermore, this method inherently possesses a standardized photography process and help ensuring process standardization at the image acquisition stage. In this sense, data quality can be assured, thereby having a positive impact on downstream task. However, prior to large-scale implementation, several pivotal aspects inherent to imaging devices, including imaging quality, imaging repeatability, and imaging reproducibility, warrant meticulous evaluation and address [15]. With repeatability being a cornerstone for process standardization, our primary focus thereby was directed to the question, "How is the repeatability of imaging in fully self-service fundus photography in comparison to those executed by professional ophthalmologists?" At present, evidence from real-world scenarios is lacking.

This study used traditional fundus photography performed by experienced operators as a control, and compared a number of quantitative indicators of clinical importance that can be extracted from fundus images, to verify whether fully self-service fundus photography could achieve the similar imaging repeatability as the traditional technology.

## Results

A total of 65 participants were included, with 19 men (29.23%) and 46 women (70.77%). All of the participants had no cataract. The mean age was 57.1 ± 5.5 years. Among them, 34 participants were in the fully self-service fundus photography group and 31 were in the traditional fundus photography group. There were no significant differences in age, gender, eye side, and fundus indicators between the groups (Table 1).

**Table 1:** Description of the basic information

Indicator		Fully self-service fundus photography (*n* = 34)	Traditional fundus photography (*n* = 31)	Total (*n* = 65)	*P* value
Age	Mean(SD)	57.00 (4.43)	57.23 (6.68)	57.11 (5.53)	0.87
Gender	Male n(%)	12 (35.29)	7 (22.58)	19 (29.23)	0.26
	Female n(%)	22 (64.71)	24 (77.42)	46 (70.77)	/
Eye side	right n(%)	14 (41.18)	19 (61.29)	39 (60.00)	0.84
	Left n(%)	20 (58.82)	12 (38.71)	26 (40.00)	/
Vascular fractal dimension	Mean(SD)	1.44 (0.02)	1.43 (0.03)	1.44 (0.03)	0.16
Average vascular curvature	Mean(SD)	0.00058 (0.000066)	0.00057 (0.00007)	0.00057 (0.000067)	0.59
Mean vascular curvature within 0.5–1.0PD	Mean(SD)	0.00058 (0.00012)	0.00058 (0.00011)	0.00058 (0.00011)	0.89
CRAE within 0.5PD-1.0PD	Mean(SD)	110.74 (15.56)	107.76 (18.22)	109.32 (16.82)	0.48
PRAE within 1.5PD-2.0PD	Mean(SD)	116.31 (12.9)	116.98 (15.24)	116.63 (13.96)	0.85
CRVE within 0.5PD-1.0PD	Mean(SD)	212.13 (16.82)	206.58 (21.53)	209.48 (19.26)	0.25
PRVE within 1.5PD-2.0PD	Mean(SD)	211.29 (14.57)	214.28 (25.83)	212.71 (20.6)	0.57
CAVR within 0.5PD-1.0PD	Mean(SD)	0.52 (0.07)	0.52 (0.06)	0.52 (0.07)	0.86
PAVR within 1.5PD-2.0PD	Mean(SD)	0.55 (0.06)	0.55 (0.06)	0.55 (0.06)	0.85
Optic disc area (µm^2^)	Mean(SD)	2129939.76 (331332.54)	2226441.87 (437759.48)	2175963.85 (385,737.74)	0.32
Optic disc horizontal diameter (µm)	Mean(SD)	1574.78 (146.45)	1598.37 (155.15)	1586.03 (149.95)	0.53
Optic disc vertical diameter (µm)	Mean(SD)	1716.45 (161.79)	1763.85 (194.5)	1739.06 (178.32)	0.29
Optic cup area (µm^2^)	Mean(SD)	460450.04 (213,235.94)	485536.69 (197908.11)	472414.45 (204,852.43)	0.43^*^
Optic cup horizontal diameter (µm)	Mean(SD)	741.67 (168)	752.51 (147.06)	746.84 (157.23)	0.78
Optic cup vertical diameter (µm)	Mean(SD)	745.95 (191.47)	781.69 (177.07)	763 (184.19)	0.44

^*^Using Wilcoxon signed-rank test

There was no statistical difference in either the absolute differences (Table 2), or the extents of variation of the indicators between two groups (Table 3), which suggested that the imaging repeatability of the two group was similar. In addition, the extents of variation of all indicators were below 10% in both groups, except the optic cup area, which indicated the repeatability was good.

**Table 2:** Comparison of the absolute differences between the two groups, Mean (SD)

Indicator	Fully self-service fundus photography (*n* = 34)	Traditional fundus photography (*n* = 31)	Total (n = 65)	*P* value^*^
Vascular fractal dimension	0.02 (0.01)	0.02 (0.01)	0.02 (0.01)	0.51
Average vascular curvature	0.000035 (0.000029)	0.000029 (0.000028)	0.000032 (0.000029)	0.37
Mean vascular curvature within 0.5–1.0PD	0.000053 (0.00004)	0.000059 (0.00006)	0.000056 (0.00005)	0.94
CRAE within 0.5PD-1.0PD	6.83 (5.59)	8.98 (7.69)	7.86 (6.71)	0.35
PRAE within 1.5PD-2.0PD	6.75 (5.5)	5.37 (4.04)	6.09 (4.87)	0.36
CRVE within 0.5PD-1.0PD	10.12 (10.55)	10.47 (9.27)	10.29 (9.88)	0.65
PRVE within 1.5PD-2.0PD	11.85 (8.7)	16.31 (17.64)	13.98 (13.78)	0.46
CAVR within 0.5PD-1.0PD	0.04 (0.03)	0.04 (0.04)	0.04 (0.03)	0.38
PAVR within 1.5PD-2.0PD	0.04 (0.03)	0.04 (0.03)	0.04 (0.03)	1.00
Optic disc area (µm^2^)	59,252.59 (57,117.05)	45,037.68 (41,889.9)	52,473.17 (50,555.85)	0.27
Optic disc horizontal diameter (µm)	24.19 (24.1)	20.22 (15.82)	22.3 (20.52)	0.80
Optic disc vertical diameter (µm)	28.8 (20.65)	21.12 (14.04)	25.13 (18.09)	0.14
Optic cup area (µm^2^)	55,410.38 (63,169.78)	44,722.35 (42,611.64)	50,313.02 (54,199.98)	0.82
Optic cup horizontal diameter (µm)	57.56 (72.77)	42.49 (42.72)	50.37 (60.36)	0.55
Optic cup vertical diameter (µm)	45.67 (43.48)	32.7 (29.93)	39.48 (37.91)	0.20

^*^Using Wilcoxon signed-rank test

**Table 3:** Comparison of the extent of variation of indicators between the two groups, Mean (SD)

Indicator	Fully self-service fundus photography (*n* = 34)	Traditional fundus photography(*n* = 31)	Total(*n* = 65)	*P*-value^*^
Extent of variation				
Vascular fractal dimension	1.28 (0.9)	1.19 (1.01)	1.24 (0.95)	0.57
Average vascular curvature	0 (0)	0 (0)	0 (0)	0.39
Mean vascular curvature within 0.5–1.0PD	9.2 (6.57)	10.27 (10.12)	9.71 (8.4)	0.96
CRAE within 0.5PD–1.0PD	6.38 (5.42)	8.67 (8.2)	7.47 (6.93)	0.39
PRAE within 1.5PD–2.0PD	5.77 (4.67)	4.56 (3.5)	5.19 (4.16)	0.38
CRVE within 0.5PD–1.0PD	4.77 (4.85)	5.01 (4.4)	4.88 (4.61)	0.56
PRVE within 1.5PD–2.0PD	5.65 (4.18)	7.19 (6.62)	6.38 (5.49)	0.46
CAVR within 0.5PD–1.0PD	6.9 (6.06)	9.08 (8.12)	7.94 (7.15)	0.39
PAVR within 1.5PD–2.0PD	7.63 (5.67)	7.97 (6.59)	7.79 (6.08)	0.99
Optic disc area (µm^2^)	2.82 (2.8)	2.01 (1.54)	2.43 (2.3)	0.21
Optic disc horizontal diameter (µm)	1.53 (1.51)	1.27 (0.98)	1.4 (1.28)	0.59
Optic disc vertical diameter (µm)	1.71 (1.31)	1.21 (0.83)	1.47 (1.13)	0.16
Optic cup area (µm^2^)	13.94 (16.27)	10.01 (9.86)	12.07 (13.64)	0.61
Optic cup horizontal diameter (µm)	8.33 (10.5)	5.83 (5.95)	7.14 (8.66)	0.52
Optic cup vertical diameter (µm)	6.54 (6.51)	4.5 (4.2)	5.57 (5.58)	0.27

^*^ Using Wilcoxon signed-rank test

The multivariate analysis results were consistent with results mentioned above. After adjusting for age and gender, the impacts of photography method on the extent of variation of all the indicators were not significant (Table 4).

**Table 4:** Comparison of the extent of variation of indicators between the two groups (multivariate analysis)

Indicator	Effect value^a,b^	Standard error	*P*-value
Vascular fractal dimension	− 0.0006	0.0023	0.78
Average vascular curvature	0	0	0.58
Mean vascular curvature within 0.5–1.0PD	0.0072	0.0206	0.73
CRAE within 0.5PD–1.0PD	0.0219	0.0169	0.20
PRAE within 1.5PD–2.0PD	− 0.0123	0.0102	0.23
CRVE within 0.5PD–1.0PD	0.0030	0.0113	0.79
PRVE within 1.5PD–2.0PD	0.0173	0.0134	0.20
CAVR within 0.5PD–1.0PD	0.0220	0.0174	0.21
PAVR within 1.5PD–2.0PD	0.0058	0.0149	0.70
Optic disc area (µm^2^)	− 0.0075	0.0056	0.18
Optic disc horizontal diameter (µm)	− 0.0025	0.0032	0.42
Optic disc vertical diameter (µm)	− 0.0044	0.0026	0.09
Optic cup area (µm^2^)	− 0.0484	0.0326	0.14
Optic cup horizontal diameter (µm)	− 0.0305	0.0207	0.14
Optic cup vertical diameter (µm)	− 0.0240	0.0133	0.07

^a^Adjusting age and gender

^b^Effect value = Traditional fundus photography group—fully self-service fundus photography group

**Fig. 1:**
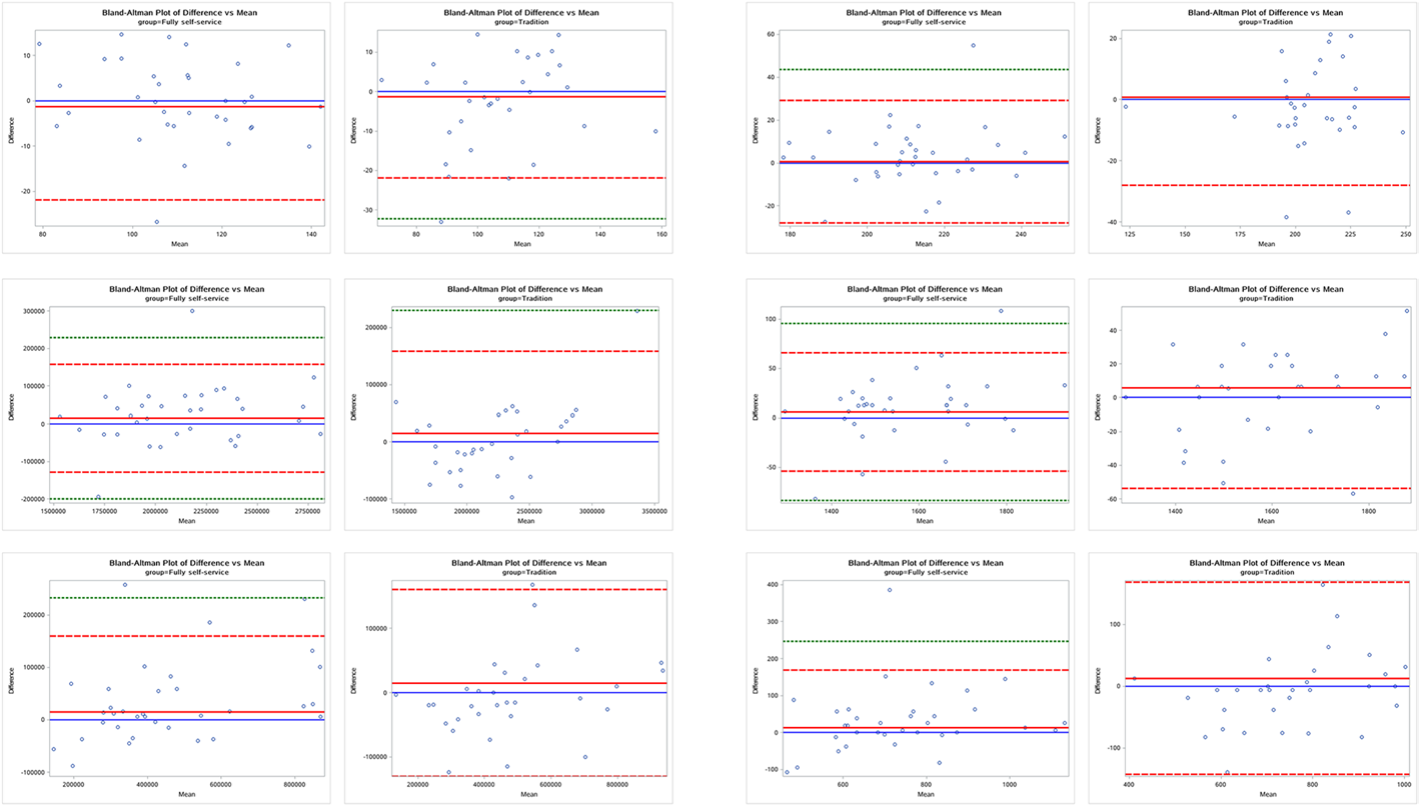
Bland–Altman plots for key measurement indicators. Top-left: CRAE within 0.5PD-1.0PD; Top-right: CRVE within 0.5PD-1.0PD; Medium-left: Optic disc area; Medium-right: Optic disc horizontal diameter; Bottom-left: Optic cup area; Bottom-right: Optic cup horizontal diameter. Red dashed line: ± 2SD reference lines; Green dashed line: ± 3SD reference lines; Red solid line: Absolute difference; Blue solid line: Zero reference line

The Bland–Altman plots depicted in Fig. 1 indicate that for crucial measurement indicators such as CRAE and CRVE, over 95% of the absolute difference records were positioned within the ±2SD reference lines for both groups. The mean values of the absolute difference closely aligned with the zero-reference line. This pattern held true for other parameters including optic disc area, optic disc horizontal diameter, optic cup area, optic cup horizontal diameter and others (see in Additional file 4: Figs. S2–S10). Consequently, these plots substantiate that the repeatability of both photography methodologies was satisfactory.

## Discussion

This study, through a comparative analysis of the performance of two unique fundus cameras in real-world eye disease screening situations, unveiled that the image repeatability of fully self-service fundus photography is comparable to that of traditional fundus photography performed by professionals. No statistical difference was noted in the absolute differences between various photography utilizing self-service devices, with the degree of variation for most indicators staying under 10%. These preliminary findings indicate that the technology underpinning fully self-service fundus photography is approaching maturity, promising to enhance the efficiency and reach of large-scale eye disease screening programs.

Since the first imaging of the retina in 1886 [16], there has been a continuous endeavor to achieve higher quality imaging of the retina to aid in disease diagnosis, leading to the development of a variety of fundus cameras. The emergence of the first digital camera in 1975 propelled the transition of medical records from analog to digital [17], and nonmydriatic digital fundus cameras have found successful adoption in or even outside the realm of ophthalmology clinic settings [18–20]. Currently, an increasing number of fully self-service fundus cameras are being utilized in practice. The major distinction between fully self-service fundus photography and traditional fundus photography lies in the device operator. Traditional fundus cameras necessitate operation by professionally trained medical personnel. On the other hand, the fully self-service fundus camera is engineered to utilize eye structure recognition, pressure sensors, voice prompt systems, among other technologies, to facilitate a thoroughly automated shooting process. Therefore, it is more suitable for community-based eye disease screening, especially in settings short of professionals. Additionally, this may favor non-ophthalmologist physicians who hope to use fundus images for screening other systematic diseases, as they usually do not perform fundus photography with assurance.

In terms of the measurement indicators used in our study, commonly used indicators including vascular fractal dimension [21], and optic disc and macular position [22] were used. To expand the focus on different areas within the images, we also included an expanded array of indicators encompassing vascular bending rate [23–25], retinal arteriovenous ratio [26], and mean arterial diameter [27]. This allows for a more comprehensive comparison in our research. Additionally, all measurement indicators were objectively derived from fundus images using a verified AI-driven image quantitative analysis software [28]. This method helps in diminishing the bias that may be induced by manual measurement processes.

Unlike the fully self-service fundus camera used in our study, the fully self-service fundus photography in the preceding study was primarily facilitated through the integration of smartphones and various aids [29–31]. Their approach basically involved the introduction of an additional lens in alignment with the smartphone, coupled with customized software that enables the capture, annotation, and secure transmission of fundus images. It represents a convenient type of non-medical device-based imaging modality that can be operated by non-specialists. However, certain studies suggest that the quality of smartphone imaging is inferior to that of traditional fundus imaging, because it is difficult to obtain clear images without mydriasis in a short time [32]. Modern fundus cameras can utilize confocal scanning laser technology to achieve wide-angle photography without pupil dilation, making them more suitable for full automation. Compared to previous study, the fully self-service fundus camera used in our study, while compromising a degree of convenience, can achieve high-quality images similar to those captured by ophthalmologist-operated fundus cameras. The quality of retinal images directly affects the accuracy of interpretation results, especially within the realm of AI-assisted diagnostic models [33, 34]. Moreover, this approach inherently possesses a standardized photography process, thereby providing a high-quality standardized data basis for relevant AI research.

Overall, this study presents multiple strengths. First and foremost, it was conducted in real-world community-based fundus disease screening scenarios rather than in laboratory settings, ensuring the results genuinely represent the stability of fully self-service cameras in practical deployments. Secondly, as highlighted earlier, all the indicators utilized in this study were measured employing validated AI technology. Thus, the reliability reported in this study is solely attributable to the fundus photography technology.

Nonetheless, certain limitations persist. Similar to a preceding study [35], the sample size in this investigation is limited. However, the breadth of indicators evaluated, encompassing as many as 15 distinct indicators, potentially mitigates the instability in results that a smaller sample size might induce. Secondly, our study was constrained to individuals without cataracts. Future investigations should encompass populations presenting with early-stage, quantifiable cataracts to enhance the validity of our findings across a broader clinical spectrum. A more refined approach could involve randomization or employing both camera systems on each participant's eye.

## Conclusion

This study is one of the first to verify the repeatability of fully self-service fundus photography in community-based screening, thereby providing supportive evidence for the promotion of this new technology. The results suggested the fully self-service fundus photography could achieve a good image repeatability. The findings indicate that fully self-service fundus photography can attain commendable image repeatability. Therefore, within the scope of community-driven eye disease screening, the incorporation of fully self-service fundus photography is a highly valuable option.

## Method

### Study design

This study, set within the framework of the ongoing Shanghai Digital Eye Diseases Screening Program (SDEDS) initiated in 2010, is a cross-sectional examination conducted in Shanghai, China. Since 2010, a remote ophthalmology-based eye disease screening initiative has been launched in Shanghai, wherein residents can avail free routine ophthalmic examinations at community health service centers. Residents exhibiting a visual acuity less than 4.7 are further photographed with a 45° fundus image centered around the macula. Designated reading centers with retinal specialists make diagnoses based on the fundus images, after which screening results are relayed back to the community health service centers, where patients receive consultation and medical advice from general practitioners. Nowadays, about 100 thousand residents receive free routine eye examinations at 249 community health service centers every year, leading to the formation of the Shanghai Resident Eye Health Information Service System. This system encompasses common blinding eye diseases such as AMD, DR, and high myopia. To maximize the efficiency of the Shanghai program, community health service centers have coordinated specific locations and times, and individuals diagnosed with relevant eye diseases in hospitals can still participate in the free annual community screenings to monitor disease progression.

The research procedure unfolds through three main steps. In the first step, we collected fundus color photographs from community residents using two varied fundus cameras, in accordance with the working sites of the SDEDS. Following that, we used AI-driven image quantitative analysis software to extract clinically relevant indicators from the fundus images. Finally, a statistical analysis was performed on the relevant indicators derived from the images by both imaging techniques, to validate whether fully self-service fundus photography could reach imaging repeatability comparable to traditional methodologies.

### Data acquisition

This study adhered to the Declaration of Helsinki and was approved by the Institutional Review Board of the Shanghai Eye Diseases Prevention & Treatment Center (2022SQ007). All fundus images and clinical data were anonymized. Informed consent was obtained from all patients.

The study was conducted in the Sijing community of Songjiang District, Shanghai. In 2022, this community procured a self-service fundus camera named Kestrel 3100 m, which is an upgraded version of their existing traditional fundus camera. Both cameras possess the same imaging system, with the upgraded new camera adding a fully automatic photography feature. Nowadays, both traditional fundus photography and self-service fundus photography are utilized at their screening stations.

Residents were recruited into this study in the May and June, 2023, coinciding with the annual community-based fundus diseases screening. Community residents coming for screening would initially use either of the mentioned fundus cameras for a routine capture. Following introduction and obtaining informed consent, researchers conducted an additional fundus capture for residents willing to participate in this study. The equipment used for the latter capture was identical to the one used by the resident in the prior capture. The time interval between the two captures did not exceed 5 min. Residents who used the fully automatic fundus photography equipment for both captures were categorized into the fully self-service fundus photography group, while those who used the traditional fundus photography equipment for both captures were categorized into the Traditional fundus photography group.

### Description of device

The fundus imaging device employed in our research is the Kestrel 3100 m, manufactured by Chongqing Beyeo New Vision Medical Equipment Co., Ltd. This non-mydriatic camera is designed to obtain clear fundus photographs through pupils as small as 2.8 mm without necessitating pharmacological dilation, thus facilitating its application across a diverse patient demographic.

**Fig. 2:**
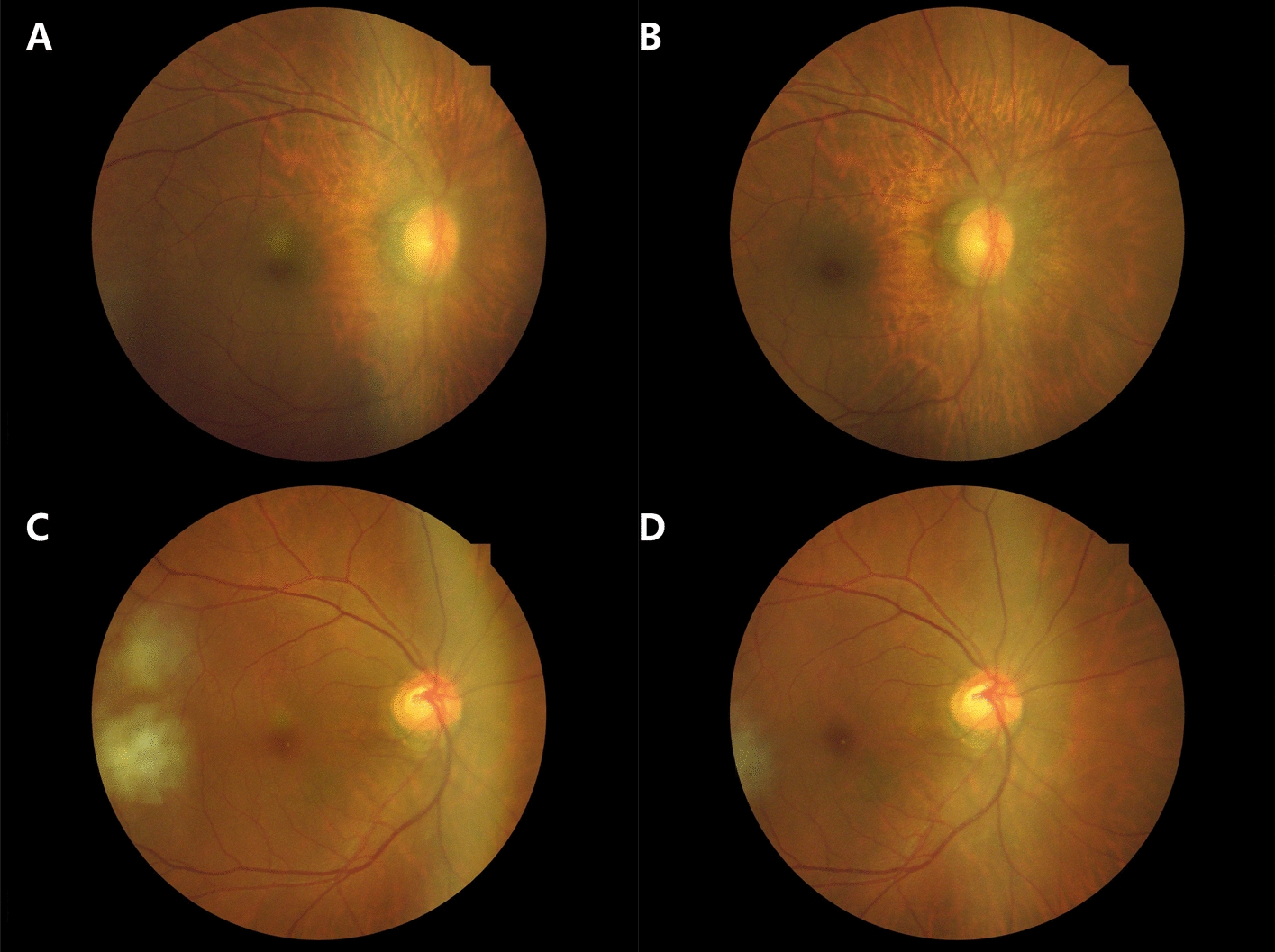
Example images captured by the Kestrel 3100 m fundus camera during community eye disease screening. **A** and **B** illustrate traditional manual photography mode images of a resident’s eye, centered on the macula and optic disc, respectively. **C** and **D** depict images from a fully self-service photography mode of another resident’s eye, also centered on the macula and optic disc, respectively

The Kestrel 3100 m supports both conventional and fully self-service fundus photography modes. In the self-service configuration, the user's involvement is minimized to positioning the patient correctly and initiating the capture process with a simple press of the “start” button. The device then autonomously completes the alignment, focusing, and captures high-resolution images for both eyes, as exemplified in Fig. 2. Comprehensive details and a demonstrative video of this process are accessible in Additional files 1 and 2, providing an in-depth overview of the system’s operation and capabilities.

### Measurements

In this study, selected quantitative indicators of clinical relevance were derived from fundus photographs. The repeatability of imaging was assessed by examining the consistency of these crucial indicators across two distinct imaging sessions. These indicators were quantified using a particular AI software (Additional file 3), referenced in a prior publication [28]. Utilizing the modified Parr-Hubbard formula [36], the diameters of the six principal retinal arteries and veins, within a range of 0.5 to 1.0 optic disc diameter from the optic disc edge, were ascertained to determine the Central Retinal Artery Equivalent (CRAE), Central Retinal Vein Equivalent (CRVE), and the Arterio-Venous Ratio (CAVR). Similarly, within a region of 1.5 to 2.0 optic disc diameter from the optic disc, the Peripheral Retinal Artery Equivalent (PRAE), Peripheral Retinal Vein Equivalent (PRVE), and Peripheral Arteriole-to-Venule Ratio (PAVR) were calculated. Moreover, Vascular Fractal Dimension, average vascular curvature within 0.5–1.0PD from the optic disc margin, along with measurements of optic disc area, optic disc horizontal diameter, optic disc vertical diameter, optic cup area, optic cup horizontal diameter, and optic cup vertical diameter were also recorded.

### Main outcomes

The primary outcome metric is defined as the absolute difference observed between two measurements of each indicator. The secondary outcome is characterized by the degree of variation between these two measurements of each indicator, formulated as: Extent of Variation = (Absolute Difference between the two measurements/Mean of the two measurements) × 100%.

### Statistical method

The two-tailed student’s *t*-test and Wilcoxon signed-rank test were employed to compare variables between two groups according to their respective distributions. The Chi-square test was utilized to compare gender and eye-side distribution among these cohorts. Multiple linear regression analysis was conducted to evaluate the impact of differing photographic methodologies on the variation of each indicator, with adjustments accounted for age and gender. To assess repeatability, a Bland–Altman plot was generated, with y-reference lines depicting the ± 2 or ± 3 standard deviations (SD) of the discrepancy between two measurements. Data analysis was performed using SAS version 9.4 (SAS Institute, Cary, NC).

### Supplementary Information

**Additional file 1. **Example video of the fully self-service fundus photography process.

**Additional file 2. **Detailed description of the fully self-service fundus camera.

**Additional file 3. **Detailed description of the AI measurement software.

**Additional file 4. **Bland–Altman plots for other measurement indicators.

## Data Availability

All data generated or analyzed during this study are included in this published article and its supplementary information files.
