# Supplementary material for: Evaluating imaging repeatability of fully self-service fundus photography within a community-based eye disease screening setting
Source: Biomed Eng Online. 2024 Mar 12;23:32. doi: 10.1186/s12938-024-01222-2 (PMC10935825; doi:10.1186/s12938-024-01222-2)
Supplement: Supplementary file 2 — Additional file 2. Detailed description of the fully self-service fundus camera. [file 12938_2024_1222_MOESM2_ESM.docx]

**Supplementary File 2: Detailed description of the fully self-service fundus camera**

The fully automatic fundus camera used in this study is the Kestrel 3100 m, produced by Chongqing Beyeo New Vision Medical Equipment Co., Ltd. In 2018, the firm developed and fabricated a fully automatic fundus camera, which subsequently obtained market clearance, distinguishing it as the inaugural device of its category to secure such authorization within China. The detailed parameters are shown in table S1.

**Table.S1 Parameter of the Kestrel 3100 m fundus camera**

| Size | 280x559x460 (mm) |
| --- | --- |
| Weight | 21kg |
| Capture angle | 50° |
| Working distance | 11mm |
| Minimum pupil | ≥2.8mm |
| Refractive compensation | ±30D |
| Pixel | 24 million |
| Positioning technology | Dual-camera positioning |
| DICOM 3.0 | support |

Traditional fundus cameras employ split-image focusing. In the realms of photography and optics, this focusing technique typically utilizes a split prism to generate two or more images. When these images are correctly focused, they align seamlessly. This method necessitates manual focusing, requiring the operator to manually adjust the alignment of the split lines to achieve precise focusing. In contrast, the fully automatic fundus camera incorporates paraxial auxiliary focusing. The term "paraxial" typically refers to directions parallel to the optical or central axis. Paraxial auxiliary focusing might involve the use of an auxiliary optical path aligned parallel to the primary one to aid in focusing. This approach can be leveraged for high-precision focusing, especially in certain intricate optical measurement or experimental apparatuses.

The device incorporates specific control methods for various image quality assessment metrics. The integrated software algorithms enable precise judgment at the pixel level and adaptively adjust based on real-time feedback. This ensures the optimization of parameters such as alignment, focus, and exposure selection, guaranteeing that image contrast, clarity, and other imaging indicators meet the required standards. The technical details related to this have undergone stringent factory tests and inspections by market regulatory authorities. However, a comparison with professional ophthalmic examination operators is still lacking.

Additionally, given its nature as a non-mydriatic instrument, regulating light exposure becomes imperative, considering the inherently constricted size of the pupil. By employing advanced optical techniques such as gentle exposure, the device effectively minimizes pupil constriction post-exposure, ensuring almost identical image quality when consecutively capturing the same fundus images of the same patient.
