# Supplementary material for: Evaluating imaging repeatability of fully self-service fundus photography within a community-based eye disease screening setting
Source: Biomed Eng Online. 2024 Mar 12;23:32. doi: 10.1186/s12938-024-01222-2 (PMC10935825; doi:10.1186/s12938-024-01222-2)
Supplement: Supplementary file 3 — Additional file 3. Detailed description of the AI measurement software. [file 12938_2024_1222_MOESM3_ESM.docx]

**Supplementary File 3: Detailed description of the AI measurement software**

Initially, fundus photographs undergo a comprehensive preprocessing protocol: regions of interest (ROI) are delineated, followed by image enhancement techniques such as denoising, normalization, and contrast amplification to accentuate vascular details. These refined images are then fed into a deep learning architecture, ResNet101-UNet, designed for precise vascular segmentation. Distinguishing between retinal arterioles and venules is achieved by analyzing attributes such as color, luminance, and the vascular network's spatial relationships. Subsequent steps involve morphological processing to extract the vessel's centerline, facilitating the measurement of vessel diameter at designated points through an innovative method where orthogonal tangents intersect the vessel's boundary. This process is depicted in Figure S1A. Moreover, the integration of the deep learning model with a visual attention-based edge detection algorithm enables the precise delineation of the optic disk. The optic disk diameter is inferred from the smallest encompassing circle within the segmented region, serving as a benchmark for calculating the diameters of nearby vascular structures.

Measurement of blood vessel tortuosity was processed as follows: The average curvature of the points on the centerline of the blood vessel was calculated as the average tortuosity of the blood vessel (Figure S2B), and the calculation was based on the following formulas. The average tortuosity of all blood vessels in the region 0.5-1PD from the optic disc margin was calculated. $C_{A}$ is the curvature of A on the center line, points B and C are located on both sides of point A, and the distance on the line from point A is equal. $R_{A}$ is the radius of the circumcircle of the △ABC formed by the three points A, B, and C. In addition, a is the side length of the opposite side of ∠A.


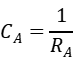

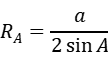


Figure S1A showcases the measurement of retinal blood vessel calibers. Retinal photographs were captured by a 45°digital retinal camera. The blue lines represent for the venules and the red lines represent for the arterioles. Six arteriolar vessels and six venular vessels with the largest average diameter were selected, and the central retinal artery equivalent (CRAE) and central retinal vein equivalent (CRVE) were calculated.

In Figure S1B, the tortuosity of retinal blood vessel is measured. The average curvature of the points on the centerline of the blood vessel was calculated as the average tortuosity of the blood vessel. The average tortuosity of all blood vessels in the region 0.5-1PD from the optic disc margin was calculated.


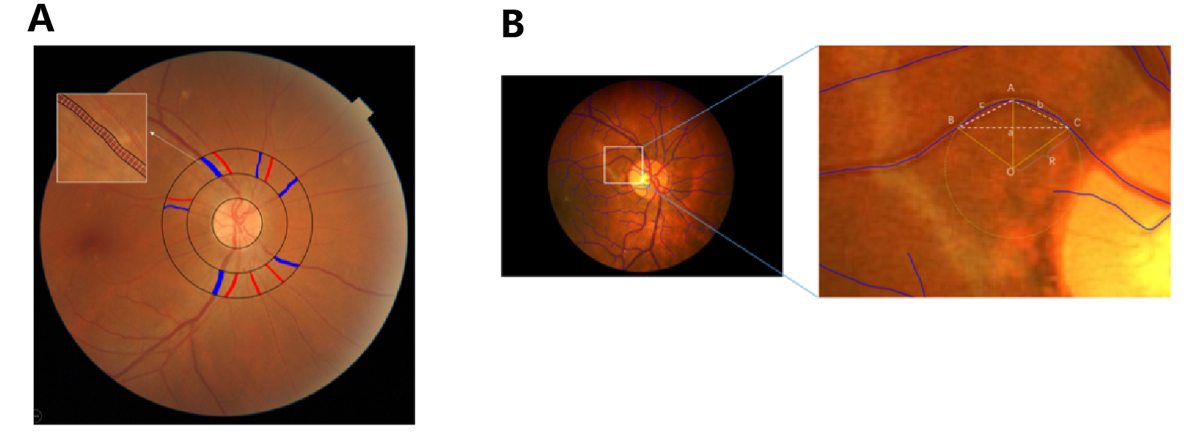


Figure S1. An example of retinal blood vessel calibers and tortuosity measurement using full-automated deep-learning based program.
