# Supplementary material for: Evaluating imaging repeatability of fully self-service fundus photography within a community-based eye disease screening setting
Source: Biomed Eng Online. 2024 Mar 12;23:32. doi: 10.1186/s12938-024-01222-2 (PMC10935825; doi:10.1186/s12938-024-01222-2)
Supplement: Supplementary file 4 — Additional file 4. Bland–Altman plots for other measurement indicators. [file 12938_2024_1222_MOESM4_ESM.docx]

**Supplementary File 4: Bland-Altman plots for other measurement indicators**

**
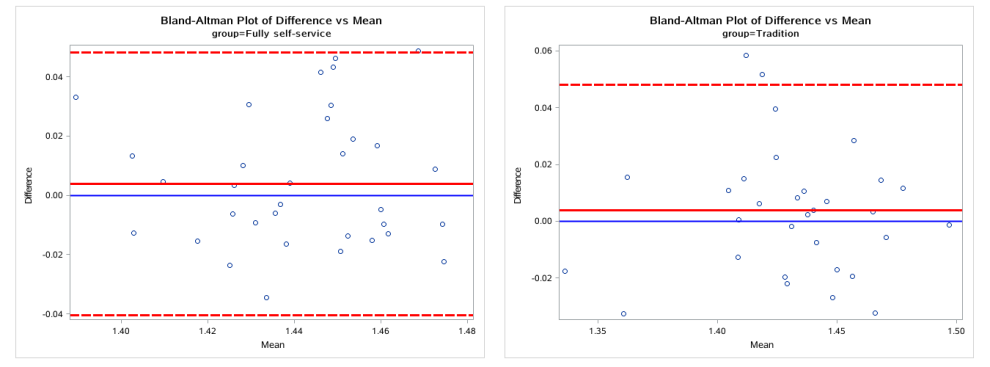
**

**Figure S2. Vascular fractal dimension**

**
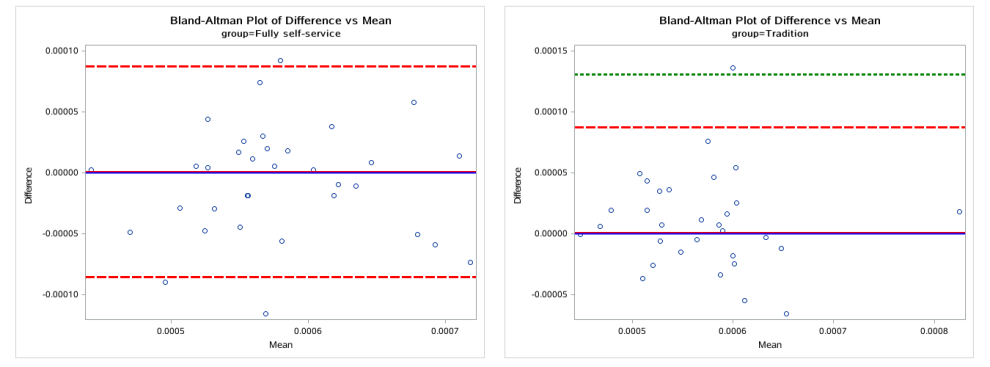
**

**Figure S3. Average vascular curvature**

**
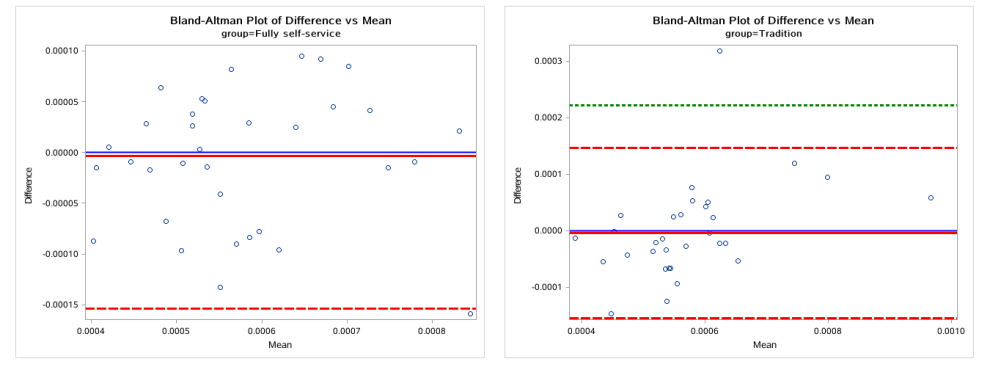
**

**Figure S4. Mean vascular curvature within 0.5-1.0PD**

**
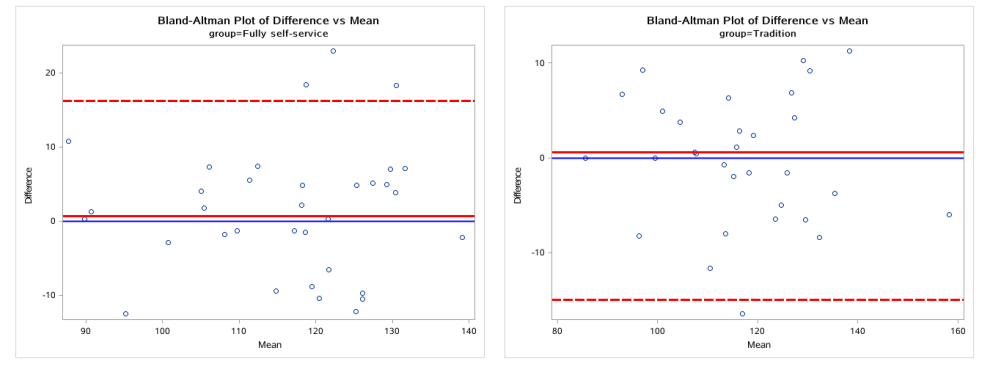
**

**Figure S5. PRAE within 1.5PD-2.0PD**

**
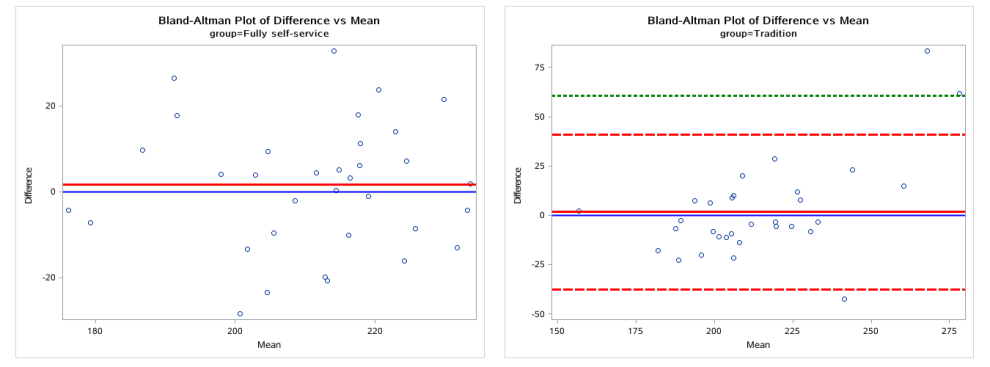
**

**Figure S6. PRVE within 1.5PD-2.0PD**

**
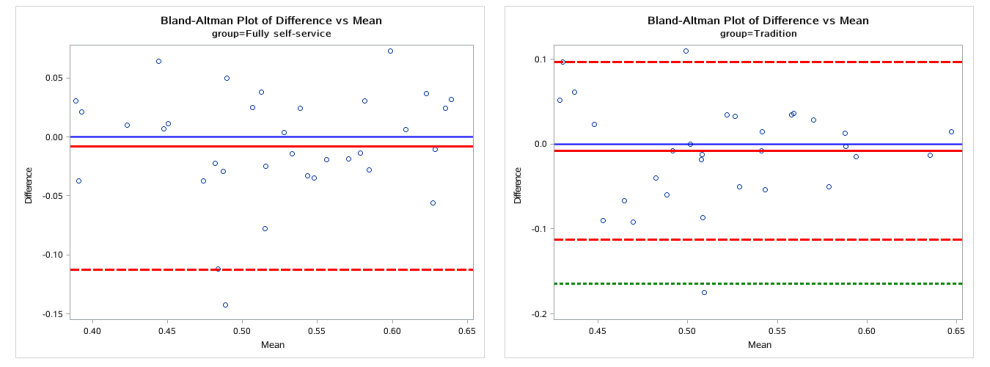
**

**Figure S7. CAVR within 0.5PD-1.0PD**

**
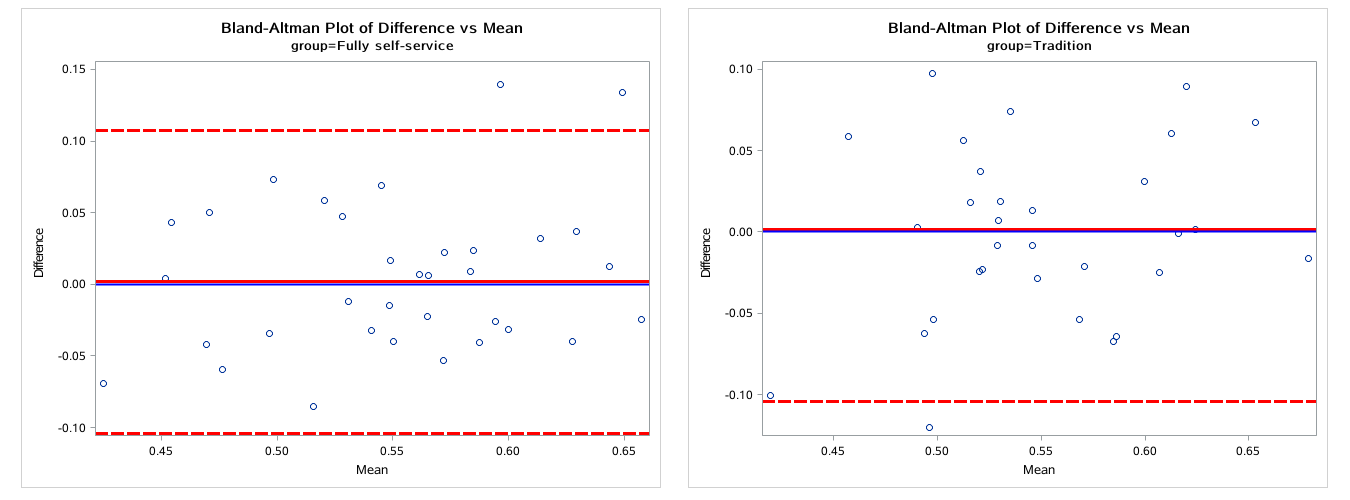
**

**Figure S8. PAVR within 1.5PD-2.0PD**

**
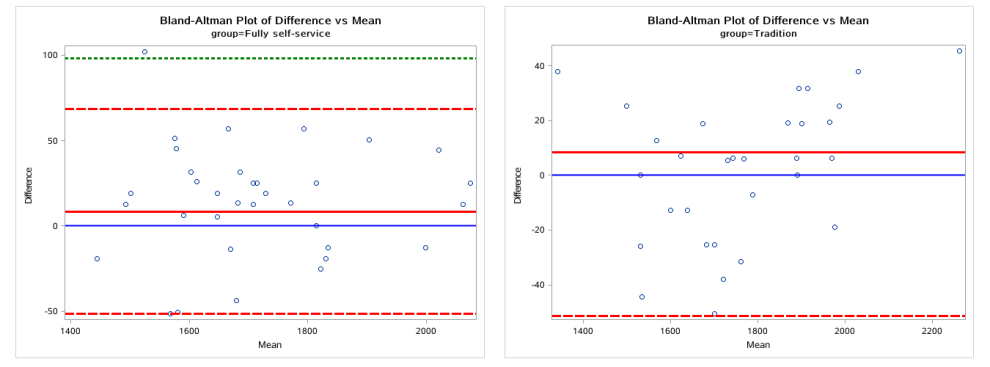
**

**Figure S9. Optic disc vertical diameter(µm)**

**
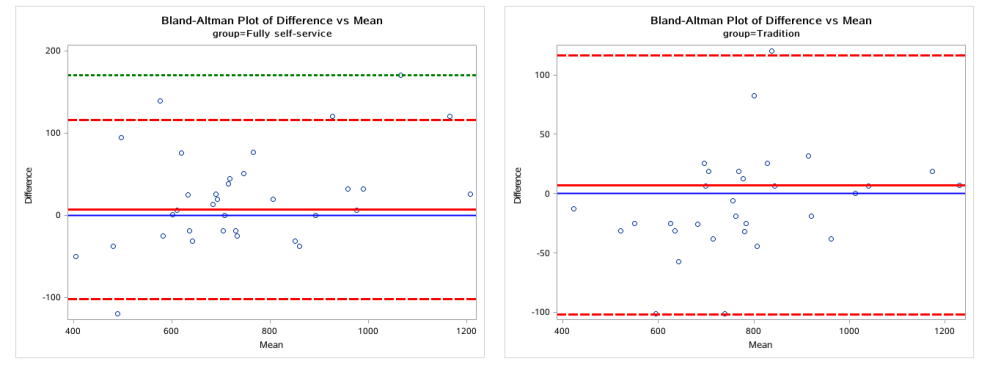
**

**Figure S10. Optic cup vertical diameter(µm)**
